# Examining opioid prescribing trends for non-cancer pain using an estimated oral morphine equivalence measure: a retrospective cohort study between 2005 and 2015

**DOI:** 10.3399/bjgpopen20X101122

**Published:** 2020-12-02

**Authors:** Emma Davies, Bernadette Sewell, Mari Jones, Ceri J Phillips, Jaynie Y Rance

**Affiliations:** 1 PhD Research Fellow, College of Human and Health Sciences, Swansea University, Swansea, UK; 2 Advanced Pharmacist Practitioner in Pain Management, Pharmacy and Medicines Management, Cwm Taf Morgannwg University Health Board, Abercynon, UK; 3 Senior Lecturer in Health Economics, Swansea Centre for Health Economics, Swansea University, Swansea, UK; 4 Research Officer, Swansea Centre for Health Economics, Swansea University, Swansea, UK; 5 Professor of Health Economics, College of Human and Health Sciences, Swansea University, Swansea, UK; 6 Professor of Public Health, Policy and Social Sciences, College of Human and Health Sciences, Swansea University, Swansea, UK

**Keywords:** primary health care, social deprivation, cohort studies, opioid prescribing, analgestics, opioid

## Abstract

**Background:**

Over the past 20 years prescription of opioid medicines has markedly increased in the UK, despite a lack of supporting evidence for use in commonly occurring, painful conditions. Prescribing is often monitored by counting numbers of prescriptions dispensed, but this may not provide an accurate picture of clinical practice.

**Aim:**

To use an estimated oral morphine equivalent (OMEQ_e_) dose to describe trends in opioid prescribing in non-cancer pain, and explore if opioid burden differed by deprivation status.

**Design & setting:**

A retrospective cohort study using cross-sectional and longitudinal trend analyses of opioid prescribing data from Welsh Primary Care General Practices (PCGP) took place. Data were used from the Secure Anonymised Information Linkage (SAIL) databank.

**Method:**

An OMEQ_e_ measure was developed and used to describe trends in opioid burden over the study period. OMEQ_e_ burden was stratified by eight drug groups, which was based on usage and deprivation.

**Results:**

An estimated 643 436 843 milligrams (mg) OMEQ_e_ was issued during the study. Annual number of prescriptions increased 44% between 2005 and 2015, while total daily OMEQ_e_ per 1000 population increased by 95%. The most deprived areas of Wales had 100 711 696 mg more OMEQ_e_ prescribed than the least deprived over the study period.

**Conclusion:**

Over the study period, OMEQ_e_ burden nearly doubled, with disproportionate OMEQ_e_ prescribed in the most deprived communities. Using OMEQ_e_ provides an alternative measure of prescribing and allows easier comparison of the contribution different drugs make to the overall opioid burden.

## How this fits in

It is known that opioid prescribing has increased in the UK over the past 20 years. Measures of prescribing vary and are not always reflective of what is seen in practice, nor do they allow easy identification of populations or individuals most at risk. This study used an OMEQ_e_ to standardise prescribing data. It demonstrated anomalies in prescription numbers and opioid burden. The use of OMEQ provides more easily comparable data across a range of opioid medicines and warrants consideration as a standard measure of prescribing.

## Introduction

The number of prescriptions for opioid medicines issued in the UK has increased substantially over the past 20 years.^1–6^ In particular, prescriptions for ‘strong’ opioids, such as morphine, oxycodone, and fentanyl, have seen greater increases than those classed as ‘weak’, such as codeine and dihydrocodeine.^1,2,6^ Prescribing continued to increase even when evidence to support using these medicines for people living with non-cancer pain is largely absent.^7–11^

National and international concerns have focused on strong opioids.^12–14^ However, dose and duration of use are more likely indicators of harm or potential for dependence than the choice of drug itself.^11,15–20^ It has been estimated that adverse events occur in as many as 78% of people using opioids over extended periods of time.^11–13^ Higher doses^14–17^ have been associated with depression and anxiety,^18–20^ and an increased risk of dependence and misuse.^21–24^ It has been proposed the burden or risk of opioids would be more accurately discussed in mg doses or dose equivalents, rather than number of prescriptions alone.^2,25^

An accurate estimation of opioid burden and risk is especially important in areas of high socioeconomic deprivation, which are associated with poorer health outcomes, higher incidence of chronic pain,^2,26,27^ and mental health disorders compared with the general population.^28^ Deprivation is associated with higher prescribing of potentially dependence-forming medicines, including opioids, especially for chronic, non-cancer pain in the UK^29^ and internationally.^30^ Furthermore, concomitant use of other medicines, such as benzodiazepines and antidepressants with opioids, have also been disproportionately reported in more deprived areas and confer additional risk of harm to the user.^31–33^

Wales has historically high levels of deprivation.^34^ In 2016, 23% of the Welsh population lived in poverty, more than in England (22%), Scotland (19%), or Northern Ireland (20%).^35^ The south of the country contains the majority of the most deprived areas in Wales,^36^ and also has the highest opioid-related death rates in England and Wales.^29^ However, only one comprehensive analysis of Welsh opioid prescribing has been undertaken.^4^

The aim of this study was to examine opioid prescribing trends in Wales between 2005 and 2015 using an estimated measure of daily OMEQ dose to standardise data. Analysis of OMEQ_e_ by deprivation quintile determined if opioid burden varied in distinct areas of socioeconomic deprivation.

## Method

### Data source

The study used individuals' anonymised data held in the SAIL databank, which is part of the national e-health records research infrastructure for Wales.^37,38^

Each individual was allocated a unique anonymised linkage field (ALF) number. The ALF allowed cross-linking between different existing datasets, providing a record of all healthcare interactions for each individual whose data is available to SAIL. A dataset was produced by cross-linking individuals’ anonymised records from the PCGP and Welsh Index of Multiple Deprivation (WIMD) 2011 datasets, based on the local super output areas (LSOAs) contained within the PCGP dataset.

At the time of this study, the databank contained complete data from 1 January 2005–31 December 2015 and so 11 years of available data were examined.

### Opioid prescriptions

Prescriptions are automatically assigned Read codes on the electronic patient record, when issued in primary care, providing consistent identification of data.^1,3,37,38^ Read codes are a thesaurus of clinical terms used to record interactions, diagnoses, and interventions in primary care settings in Wales. A list of Read codes was compiled for all prescribable oral and transdermal opioid medicines used for analgesia, including combination products, for example, paracetamol and codeine (co-codamol), using the NHS Information Authority’s clinical terminology browser. Products licensed for the management of misuse and injectable opioids, which are reserved for palliative care, were excluded.

Only data for people aged ≥18 years between 2005 and 2015 without a recorded cancer diagnosis (identified using Read codes for cancer diagnoses or treatment) at any time between 2004 and 2015 were included in the analysis.

All data were subjected to repeated cross-sectional sampling to determine prescribing trends over the study period.

### Estimated oral morphine equivalent dose

At the time of this study, dispensing data were not included within SAIL datasets. The prescribed drug product, including strength, was available from PCGP data, but not administration directions and quantity of each opioid product prescribed. Therefore, actual oral morphine equivalent dose for each individual could not be calculated.

An OMEQ_e_ measure was developed using data available from SAIL (Table 1). For each product, the recommended daily dose per day was taken from the *British National Formulary* ^39^ and electronic medicines compendium (emc).^40^ The daily dose was converted to a daily OMEQ_e_ value, based on available conversion tables.^8,39^ Daily OMEQ_e_ for each product was multiplied by the number of prescriptions issued each year to determine annual totals (Table 1). Results were stratified by drug, with less frequently prescribed medicines (oral diamorphine, dipipanone, hydromorphone, meptazinol, methadone tablets, pentazocine, pethidine, and tapentadol) grouped as ‘other’ opioids.

**Table 1.** Example of calculations for OMEQ_e_ (mg) using 2005 data for female subjects

	Units used for calculating annualised OMEQ_e_			
Drug product	Recommended daily dose^a^ ^,b^	Oral morphine equivalent of daily dose (mg)^c^ ^,d^	Annual number of prescriptions	Annualised total OMEQ_e_ burden (mg)
**Buprenorphine**				
10 mcg per hour	1 patch per week	24	28	672
52.5 mcg per hour	1 patch twice a week	126	354	44 604
**Codeine**				
Co-codamol 8/500	2 tablets 4 times a day	6.4	17 952	114 893
Codeine phosphate 30 mg	2 tablets 4 times a day	24	16 293	391 032
Zapain capsules (30/500)	2 tablets 4 times a day	24	112	2688
**Dihydrocodeine**				
Co-dydramol 10/500	2 tablets 4 times a day	8	153 047	1 224 376
DHC Continus 90 mg MR tablet	1 tablet twice a day	18	1009	18 612
Remedeine tablet	2 tablets 4 times a day	16	1295	20 720
**Fentanyl**				
Durogesic 100 mcg per hour patch	1 patch every 3 days	360	131	47 160
Fentanyl 200 mcg SL lozenge	1 lozenge 4 times a day	120	40	4800
Fentanyl 25 mcg per hour patch	1 patch every 3 days	90	3429	308 610
**Morphine**				
Morphgesic SR 10 mg m/r tablet	1 tablet twice a day	20	73	730
MXL 60 mg m/r capsule	1 capsule once a day	60	23	1380
Oramorph 10 mg/5 ml liquid 100 ml	5 mL every 2 hours	120	573	68 760
Sevredol 20 mg tablet	1 tablet every 6 hours	120	299	35 880
**Oxycodone**				
Longtec 20 mg m/r tablets	1 tablet twice a day	80	1	80
Oxycodone HCl 20 mg capsule	1 capsule every 4 hours	240	250	60 000
OxyContin 80 mg m/r tablet	1 capsule twice a day	320	262	83 840
**Tramadol**				
Dromadol XL 200 mg m/r tablet	1 tablet once daily	20	11	220
Tramadol 50 mg capsule	2 capsules 4 times a day	40	93 918	3 756 720
Tramacet 325 mg/37.5 mg	2 tablets 4 times a day	30	4450	133 500
**Other**				
Co-proxamol 32.5 mg/325 mg tablet	2 tablets 4 times a day	26	82 015	2 132 390
Hydromorphone HCl 1.3 mg capsule	1 capsule every 4 hours	58.5	6	351
Pethidine HCl 50 mg tablet	1 tablet every 4 hours	30	2381	71 430

Annualised total = estimated oral morphine equivalent of daily dose x annual number of prescriptions. Process repeated for each drug product and totalled for each year. ^a^ ^39^ ^b^ ^40^ ^c^ ^8^ ^d^ ^64^ OMEQ_e_ = estimated oral morphine equivalent.

### Measuring utilisation

The number of prescriptions and number of patients per year were calculated per drug in repeat cross-sections for each year and further stratified by deprivation quintile. Data were standardised to annual population size for the SAIL databank, using data from the Office for National Statistics (ONS)^41^ and StatsWales.^42^ Deprivation data were adjusted by each quintile’s annual population.^42^

### Data analysis

Data were extracted from the study tables within SAIL using Structured Query Language (SQL) code. Percentage change rate of number of prescriptions issued and number of people receiving prescriptions over the study period were also noted. Data were stratified into eight drug groups.

Shapiro-Wilk calculations showed data were non-parametric. Therefore, Kruskal-Wallis tests were used to examine differences in mean prescribing over the study period in the different drug groups and deprivation quintiles. Statistical analysis was conducted using IBM SPSS Statistics software (version 25.0) and figures drawn using Excel (version 16.30; retrieved from https://office.microsoft.com/excel).

### Deprivation scores

The WIMD is the official measure used by Welsh Government to determine relative deprivation of areas within Wales.^36^ The WIMD is a weighted total score of deprivation based on income (23.5%), employment (23.5%), health (14%), education (14%), geographical access of services (10%), community safety (5%), physical environment (5%), and housing (5%). Scores are not linear, so areas in group two are not twice as deprived as those in group four. Indices are published every 3 years.^43^ The 2011 index was recommended by SAIL for use in this study, as representative of the full 11-year period. There were no significant changes in LSOA or WIMD areas in that time. Data are presented in quintiles, with WIMD1 being the most deprived areas and WIMD5 the least deprived.

## Results

Prescribing data were extracted from 345 PCGPs across Wales. A total of 22 641 424 prescriptions for opioids were included in the analysis. Between 2005 and 2015, opioid prescriptions increased by 44% from 692 to 994 prescriptions per 1000 population annually. The total daily OMEQ_e_, issued from all included practices in Wales, more than doubled in the 11 years examined, from 37 662 651 mg to 76 428 768 mg. When adjusted to population, annualised daily OMEQ_e_ per 1000 population increased by 95% (from 16 266 mg to 31 665 mg) over the study period (Table 1).

### Total estimated oral morphine equivalent prescribed

**Figure 1.**
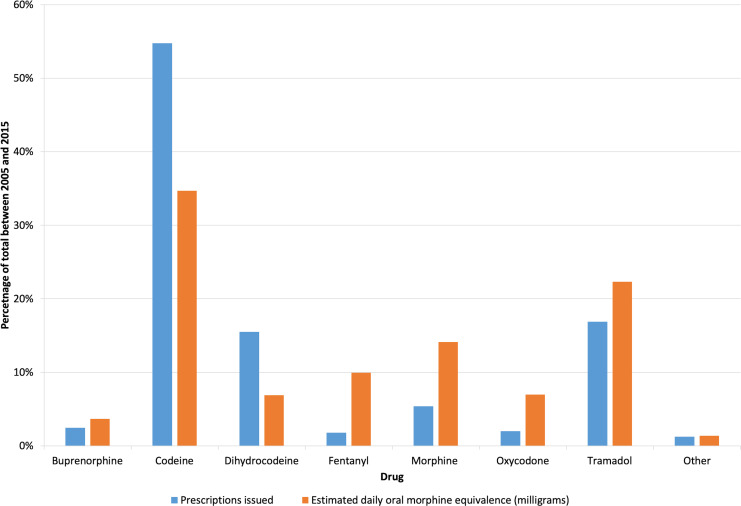
Comparison of the percentage contribution of each opioid prescribed by total prescriptions issued and total daily OMEQ_e_ dose (mg) in Wales between 2005 and 2015

**Figure 2.**
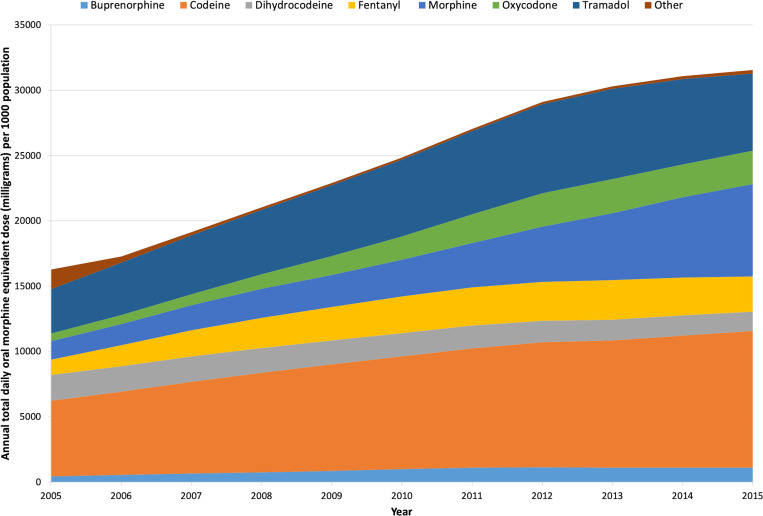
Trends in opioid prescribing across Wales, 2005–2015. Annual daily OMEQ_e_ in mg per 1000 population, stratified by drug

Codeine was the most commonly prescribed opioid (Table 2), with just under 12.5 million prescriptions issued and the highest annual total OMEQ_e_ prescribed for the study duration (Figure 1). Codeine OMEQ_e_ per 1000 population increased by 79%, from 5916 mg to 10 581 mg. Tramadol was the second most commonly prescribed opioid in Wales with a 74% increase, from 3397 mg to 5905 mg OMEQ_e_ per 1000 population, although annual total OMEQ_e_ started to reduce from 2014 (Figure 2).

**Table 2.** Daily OMEQ_e_ (mg) issued on prescription, given as annual totals and adjusted to population, stratified by drug

	Oral or transdermal opioids			
	Total daily OMEQe (mg) dose prescribed	Annual total daily OMEQe dose (mg) per 1000 population	OMEQe dose (mg) per prescription issued	Annual number of prescriptions issued per 1000 population
					**Buprenorphine**	23 641 528			
2005	977 464	422	98	4
2015	2 756 458	1142	37	31
Rate change, %	182	170.5	–61.7	606.3
**Codeine**	223 817 156			
2005	13 743 115	5916	17	357
2015	25 593 382	10 581	19	549
Rate change, %	86.2	78.8	16.2	53.9
**Dihydrocodeine**	44 600 874			
2005	4 368 806	1887	12	154
2015	3 471 460	1438	13	109
Rate change, %	–20.5	–23.8	7.7	–29.2
**Fentanyl**	64 138 905			
2005	2 695 290	1164	186	6
2015	6 496 270	2691	147	18
Rate change, %	141.0	131.2	–21.2	193.2
**Morphine**	91 132 530			
2005	3 293 220	1422	86	17
2015	17 047 800	7063	68	104
Rate change, %	417.7	396.6	–20.6	525.6
**Oxycodone**	45 120 680			
2005	1 316 480	569	105	5
2015	6 165 400	2554	100	26
Rate change, %	368.3	349.3	–4.9	372.4
**Tramadol**	144 173 635			
2005	7 865 695	3397	36	95
2015	14 252 335	5905	38	156
Rate change, %	81.2	73.8	5.7	64.4
**Other**	8 888 696			
2005	3 446 735	1719	27	56
2015	699 711	347	58	5
Rate change, %	–79.7	–79.8	117.4	–91.0

Results are rounded to nearest whole number. Rate change (%) calculated using original, unrounded data. Original data are available from the authors on request.

Large increases were noted in ‘strong’ opioids (morphine, oxycodone, fentanyl, and buprenorphine) during the study (Figure 2). Morphine OMEQ_e_ increased by 397%, from 1422 mg to 7063 mg per 1000 population (Table 2). By 2015, morphine was prescribed at three times the equivalent dose of either oxycodone (increased 349%, from 569 mg to 2554 mg per 1000 population) or fentanyl (increased 131%, from 1164 mg to 2691 mg per 1000 population).

Overall, 71% of the total opioid burden in the areas of Wales covered by the SAIL databank was accounted for by three drugs: codeine (35%), tramadol (22%), and morphine (14%). Statistically significant differences were found between the 11-year total OMEQ_e_ when each drug group was compared with the others (*P*<0.001, H = 73.5, ฦ^2^ = 0.8).

### Opioid prescribing trends by deprivation

**Figure 3.**
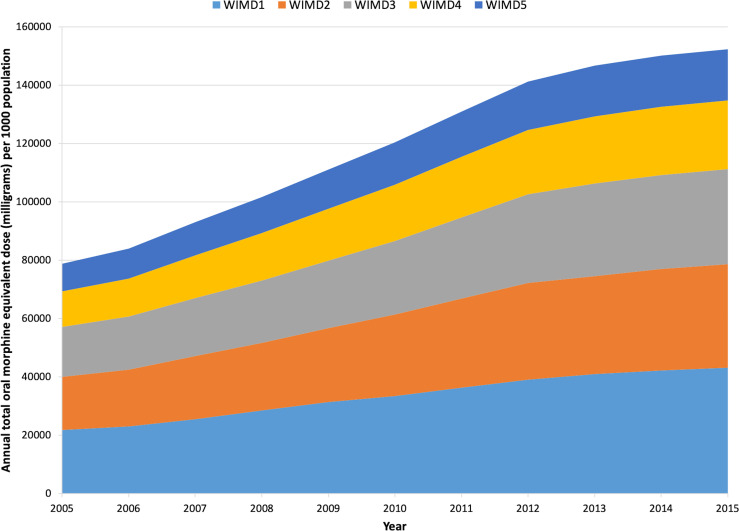
Trends in opioid prescribing across Wales, 2005–2015. Annual daily OMQE_e_ (mg) per 1000 population, stratified by deprivation. Welsh Index of Multiple Deprivation 2011 (WIMD 2011), where WIMD1 = most deprived, WIMD5 = least deprived.

Figure 3 illustrates the trends in annualised daily OMEQ_e_ of all oral and transdermal opioids stratified by the WIMD (2011). Over the study, people in the most deprived quintiles (WIMD1) were prescribed an estimated 100 711 696 mg more OMEQ_e_ than in the least deprived (WIMD5) (Table 3).

**Table 3.** Trends in OMEQ_e_ (mg) prescribing stratified by deprivation

	Oral or transdermal opioids	
Deprivation quintile	Total daily OMEQ_e_ dose (mg) prescribed	Total daily OMEQ_e_ dose (mg) per 1000 population
**WIMD1**		
2005	10 319 636	21 757
2015	21 167 919	43 176
Rate change, %	105.1	98.4
Total prescribed^a^	176 824 265	
**WIMD2**		
2005	8 590 375	18 203
2015	17 399 026	35 475
Rate change, %	102.5	94.9
Total prescribed^a^	146 459 878	
**WIMD3**		
2005	7 684 060	17 108
2015	15 342 942	32 564
Rate change, %	99.7	90.3
Total prescribed^a^	129 880 669	
**WIMD4**		
2005	5 374 595	12 242
2015	10 878 897	23 534
Rate change, %	102.4	92.2
Total prescribed^a^	93 691 687	
**WIMD5**		
2005	4 486 035	9 381
2015	8 721 170	17 557
Rate change, %	94.4	87.2
Total prescribed^a^	76 112 569	

^a^Total prescribed 2005–2015. Annual OMEQ_e_ calculated as per method and stratified by deprivation quintile (Welsh Index of Multiple Deprivation [WIMD2011], where WIMD1 = most deprived, WIMD5 = least deprived). OMEQ_e_ = estimated oral morphine equivalent.

Between 2005 and 2015, OMEQ_e_ doubled in all but the least deprived (WIMD5) areas (Table 3). Twenty-eight per cent (176 824 265 mg of 622 969 068 mg) of total OMEQ_e_ was issued in the most deprived areas of Wales. In contrast, 12% (76 112 569 mg) were prescribed in the least deprived areas. Throughout the study, OMEQ_e_ prescribed in WIMD1 areas remained more than twice those noted in WIMD5 areas (Table 3) for both total OMEQ_e_ (mg) and OMEQ_e_ per 1000 population. Despite large percentage increases in all quintiles, the difference between total OMEQ_e_ prescribed per quintile were statistically significant (*P*<0.001, H = 34.5, ฦ^2^ = 0.61).

## Discussion

### Summary

This study identified trends in opioid prescribing in Wales, similar to those previously reported in other parts of the UK.^1,3,6,26,27,44^ A marked increase in opioid burden in Wales between 2005 and 2015 was noted. Using the OMEQ_e_ measure described, opioid burden in the study population nearly doubled in 11 years. Increasing deprivation was associated with higher OMEQ_e_ and, consequently, a higher burden per person, despite rises in percentage terms being similar in all WIMD 2011 quintiles.

### Strengths and limitations

Large sets of prescribing and diagnostic data have been validated as an accurate means for conducting healthcare population research,^45,46^ as they reduce recall bias and regional variation. In this study, anyone registered with included practices and prescribed an opioid medicine were included in the analysis, avoiding selection bias. This is the first study of Welsh data to utilise OMEQ_e_ to better understand the burden of opioid prescribing on the population. Using linkage systems within SAIL datasets, data from people with a recorded cancer diagnosis could be excluded from analysis. The data confidently reflects prescribing for non-cancer pain, unlike other recent studies that assumed the majority of prescribing was attributable to persisting, non-cancer pain based on longevity of prescribing and dose forms used.^2,6^

Other studies have suggested large increases in prescribing are attributable to a range of drugs.^2,6,47^ The current study showed that three drugs were responsible for the majority of prescribing. This may, in part, be owing to the effective use of National Prescribing Indicators, which, in particular, have encouraged morphine to be used as first-line ‘strong’ opioid.^48^

Prescribing data provide an indication of intention to treat but does not confirm consumption. It also does not indicate the diagnosis or how long an individual might have been using the medication. Moreover, data presented here did not identify people receiving more than one opioid medicine and, so, would have higher individual OMEQ_e_ burdens.

It was not possible to access dispensing data, which provides details required to accurately calculate OMEQ. The authors' estimated measure (OMEQ_e_) required assumptions to be made in regard of daily dose prescribed. Also, quantity could not be verified in order to calculate duration of use. However, the trends are similar to those reported elsewhere in the UK.^1–3,6,27,44^

Further analysis is required to determine an individual’s daily intake, where multiple opioids and strengths of products are prescribed. While prescription numbers have started to stabilise or reduce since the end of the study,^6,48,49^ concerns remain about the number of people receiving supramaximal opioid doses and lengthy durations of use.^11,32^

In the study, opioid medicines were identified by Read codes and accuracy of data extraction depended on the inclusivity of the coding used. Similar rationales for deciding which opioid products to include in analysis of primary care prescribing have been adopted by other UK-based authors.^1,3,6,27^ However, incomplete coding lists could result in an under-representation of prescribing.

### Comparison with existing literature

Examining trends by prescription numbers alone is likely to underestimate the opioid burden within a population. Using English data, Curtis *et al* demonstrated a 34% growth in prescription numbers equated to a 127% increase in OMEQ burden between 1998 and 2016.^6^ In the present study, a 44% increase in prescription numbers in Wales, translated into a 95% increase in opioid burden using the OMEQ_e_ measure described.

Another measure of prescribing is defined daily doses (DDD), devised by the World Health Organization:^50^ DDDs are *‘* *the assumed average maintenance dose per day for a drug used for its main indication in adults* *.* *’* However, DDDs vary for each drug and between formulations of the same drug.^50^ When OMEQ was used to compare prescribing in four Nordic countries, it demonstrated noteworthy differences in patterns of opioid consumption compared with those seen with DDDs.^43^ ‘Weak’ opioids, such as codeine, carry higher DDD values than ‘strong’ opioids like morphine. Countries where codeine predominated, appeared to have high overall opioid prescribing, which was reversed when OMEQ was used and the contribution of ‘strong’ opioids accounted for.^43^

Prescribers’ understanding of OMEQ is poor.^51–53^ Use of OMEQ as a measure of prescribing might improve comprehension of opioid equivalence and lead to safer prescribing.

Substantial increases in opioid prescribing, with higher levels in more deprived populations, were also reported in other parts of the UK^2,27^ and internationally.^54–57^ Increased levels of prescribing in areas of high socioeconomic deprivation has been linked to greater reported pain intensity.^26^ However, limited evidence supports the notion that opioids are effective at reducing pain, particularly in the longer term.^8,58,59^ High-dose opioids (above 120 mg OMEQ) have been associated with increased levels of pain.^60,61^ In the context of this and previous studies,^2,6,26,27^ the implications of increased opioid prescribing in more deprived areas are concerning. It exposes the most vulnerable people to higher levels of medicines, which may be ineffective at best, and could cause additional health and well-being complications.^11^

### Implications for practice

OMEQ is a useful measure of opioid utilisation in the general population and an individual basis.^25^ This study has demonstrated differences between assumed burden of opioid prescribing using OMEQ_e_ and prescriptions issued, which might have important clinical implications. Evaluating opioid prescribing using OMEQ would provide easily comparable data that better reflects clinical practice. Reasons for disparities in opioid burden between areas of deprivation need further investigation. Lack of availability and acceptability of non-pharmacological management and services have been suggested among reasons why prescribing is favoured.^62,63^ Use of OMEQ as a measure of opioid burden should be considered as a means of identifying ‘at risk’ populations and individuals, as prescription numbers reduce.
